# Radiosensitization of mammary carcinoma cells by telomere homolog oligonucleotide pretreatment

**DOI:** 10.1186/bcr2639

**Published:** 2010-09-16

**Authors:** Desheng Weng, Monique C Cunin, Baizheng Song, Brendan D Price, Mark S Eller, Barbara A Gilchrest, Stuart K Calderwood, Jianlin Gong

**Affiliations:** 1 Department of Medicine, Boston University School of Medicine, 72 E Concord St, Boston, MA 02118, USA; 2 Department of Radiation Oncology, Dana-Farber Cancer Institute, Harvard Medical School, 44 Binney St, Boston, MA 02115, USA; 3 Department of Dermatology, Boston University School of Medicine, 72 E Concord St, Boston, MA 02118, USA; 4 Molecular and Cellular Radiation Oncology, Beth Israel Deaconess Medical Center, Harvard Medical School, 330 Brookline Avenue, Boston, MA 02215, USA

## Abstract

**Introduction:**

Ionizing radiation (IR) is a widely used approach to cancer therapy, ranking second only to surgery in rate of utilization. Responses of cancer patients to radiotherapy depend in part on the intrinsic radiosensitivity of the tumor cells. Thus, promoting tumor cell sensitivity to IR could significantly enhance the treatment outcome and quality of life for patients.

**Methods:**

Mammary tumor cells were treated by a 16-base phosphodiester-linked oligonucleotide homologous to the telomere G-rich sequence TTAGGG (T-oligo: GGTTAGGTGTAGGTTT) or a control-oligo (the partial complement, TAACCCTAACCCTAAC) followed by IR. The inhibition of tumor cell growth *in vitro *was assessed by cell counting and clonogenic cell survival assay. The tumorigenesis of tumor cells after various treatments was measured by tumor growth in mice. The mechanism underlying the radiosensitization by T-oligo was explored by immunofluorescent determination of phosphorylated histone H2AX (γH2AX) foci, β-galactosidase staining, comet and Terminal deoxynucleotidyl transferase dUTP Nick End Labeling (TUNEL) assays. The efficacy of the combined treatment was assessed in a spontaneous murine mammary tumor model.

**Results:**

Pretreatment of tumor cells with T-oligo for 24 hours *in vitro *enhanced both senescence and apoptosis of irradiated tumor cells and reduced clonogenic potential. Radiosensitization by T-oligo was associated with increased formation and/or delayed resolution of γH2AX DNA damage foci and fragmented DNA. T-oligo also caused radiosensitization in two *in vivo *mammary tumor models. Indeed, combined T-oligo and IR-treatment *in vivo *led to a substantial reduction in tumor growth. Of further significance, treatment with T-oligo and IR led to synergistic inhibition of the growth of spontaneous mammary carcinomas. Despite these profound antitumor properties, T-oligo and IR caused no detectable side effects under our experimental conditions.

**Conclusions:**

Pretreatment with T-oligo sensitizes mammary tumor cells to radiation in both *in vitro *and *in vivo *settings with minimal or no normal tissue side effects.

## Introduction

Breast cancer is the most common malignancy among women in the United States. It is estimated that each year 192,370 new cases will occur and 40,170 women will die from the disease (American Cancer Society Facts and Figures, 2009). Current therapies, usually in combination with surgical excision, have reduced the mortality from this disease but remain inadequate for many and may produce serious side effects. Ionizing radiation (IR) is one of the most widely used therapies but the therapeutic effect is dependent on the sensitivity of the breast cancer cells to radiation. Thus, promoting tumor cell sensitivity to IR could significantly enhance the treatment outcome and quality of life for patients. Recent studies show that telomeres are implicated in the maintenance of genomic stability and repairing of damaged DNA. Therefore, telomere-based therapy may provide a promising approach to enhancing the effect of radiotherapy and/or reducing its side effects.

Telomeres consist of guanine-rich tandem repeats that prevent chromosome ends from being recognized as DNA double-strand breaks. McClintock's historical observation that loss of telomeric sequences in maize chromosomes renders DNA ends recombinogenic highlighted the importance of telomeres and their associated complexes in chromosomal integrity [1]. More recent work has established that disruption of the T-loop by experimental DNA damage, telomere shortening or expression of a dominant-negative mutant of loop-binding factor leads to cellular apoptosis or senescence [2,3].

A novel approach to treating cancer involves harnessing innate telomere-based DNA damage responses through use of telomere homolog oligonucleotides, termed T-oligos. Like experimental disruption of the normal telomere loop structure [4], T-oligo treatment of cancer cells *in vitro *or *in vivo *leads to apoptosis and/or senescence, depending on cell type [5-9]. However, unlike treatments that disrupt the telomere loop, T-oligos do not cause digestion of the 3' telomere overhang (repeats of TTAGGG sequences) or otherwise alter endogenous chromosomes [10]. Systemic administration of T-oligos greatly reduces tumor burden in xenograft mouse models of human melanoma and breast carcinoma [6,8]. In addition, when used in combination with conventional chemotherapy to treat human lymphoma cells *in vitro *or to treat a murine B cell lymphoma in a mouse model, T-oligos reduced the dose of these toxic agents required to achieve cell killing [11]. The detailed mechanism of tumor inhibition by T-oligo is not fully elucidated. However, it is believed that the guanine (G)-rich T-oligos enhance G quadruplex formation in single-stranded telomeric DNA (repeats of TTAGGG), stall DNA replication forks and promote DNA damage responses that lead to cellular senescence and apoptosis [12]. Selective killing of malignant cells, with sparing of normal cells, likely results from the well-recognized greater sensitivity of malignant cells to replication stress, especially those with abnormalities in the breast cancer-associated gene (*BRCA*) pathway [13,14], with fatal collapse of stalled replication forks at sites of G-quadruplex formation.

In the present study, we tested the hypothesis that T-oligo treatment sensitizes tumor cells to ionizing radiation. T-oligos have been shown to inhibit growth and induce apoptosis, autophagy and/or senescence in human pancreatic, ovarian, breast cancer, melanoma, fibrosarcoma, and glioblastoma [5-9]. Our data indicate that pretreatment of mammary tumor cells with T-oligo but not a control oligo sensitizes the tumor cells to radiation *in vitro *and in an *in vivo *tumor model.

## Materials and methods

### Mice

Female C57BL/6 mice, six to eight weeks old, were purchased from Taconic Farms (Germantown, NY, USA). MMT mice were generated by breeding MUC1 transgenic (MUC1.Tg) mice with polyomavirus middle T oncogene (PyMT)-expressing MT mice that develop spontaneous mammary carcinomas [15,16]. Animals were maintained in microisolator cages under specific pathogen-free conditions. The study of mice was approved by the Institutional Animal Care and Use Committee of Boston University Medical Center.

### Oligonucleotides

A 16-base phosphodiester-linked oligonucleotide (T-oligo: GGTTAGGTGTAGGTTT) with 56% homology to the human telomere G-rich sequence [7], and a control-oligo (the partial complement, TAACCCTAACCCTAAC) were synthesized by the Midland Certified Reagent Company (Midland, TX, USA) and resuspended in H_2_0 to give a 2 mM stock solution. For the *in vitro *studies, the stock solution was diluted into culture medium, and added to cells at a final concentration of 40 μM. In all experiments, cells were given medium containing oligonucleotide once and not refed. For the *in vivo *studies, 2 mM of T-oligo and control-oligo were diluted in sterile PBS to make a 1.2 mM concentration and 50 μL of this solution was injected into each mouse.

Earlier studies of T-oligos employed 100% homologs [5-11], establishing the efficacy for telomere homologs in comparison to inactive complementary and unrelated control sequences. However, further work revealed that G-rich oligos with substantial but less than 100% homology to telomeres were also effective in activation of the DNA damage signaling pathway leading to apoptosis of malignant cells and that some were even more effective than the same length 100% homologs [7]. One of these 16-base T-oligos was selected for the present studies.

### Cell yield and counting

Primary mammary tumor cells from MMT mice were harvested and cultured in Dulbecco's Modified Eagle's Medium (DMEM) with 10% heat-inactivated fetal calf serum (FCS), 2 mM L-glutamine, 100 U/ml penicillin and 100 μg/ml streptomycin. A triplicate set of cultured cells was pre-treated with T-oligo or control-oligo at a final concentration of 0, 10, 20, 30 or 40 μM in DMEM for 24 hours, and then irradiated with 0, 3, 6, 9, or 12Gy (Cesium^137 ^source at a dose rate of 1.06 Gy/min). The cells were trypsinized and collected at 0, 24, 48, 72 and 96 hours after irradiation for cell count using a cell counter (Coulter Corp., Miami, FL, USA).

### Clonogenic survival assay

Mammary tumor cells were trypsinized to a single-cell suspension and seeded into 10 cm tissue culture dishes (1 × 10^5 ^cells/dish). After the cells were treated with 40 μM T-oligo or control-oligo for 24 hours, they were irradiated at different dose levels (0 to 8 Gy) and placed thereafter in an incubator until cells in the control groups formed multiple large clones. The colonies were stained with 0.5% crystal violet and counted. The plating efficiency (PE) and the surviving fraction (SF) were calculated by using the formula PE = (number of colonies formed/number of cells seeded) × 100% and SF = (number of colonies formed after treatment/number of cells seeded × PE) × 100%. The standard radiation survival curve was constructed and the mean lethal dose (D_0_), which represents the dose required to reduce the fraction of surviving cells to 37% (1/e) of its previous value, was calculated by fitting the data with the multitarget-single hit model and linear-quadratic model [17].

### Immunofluorescent γH2AX staining

Tumor cells plated into eight-well chambers were pretreated with T-oligo or control-oligo for 24 hours and then irradiated. After radiation, cells were fixed in 4% paraformaldehyde, and then treated with a 0.2% NP40/PBS solution for 15 minutes at room temperature. Cells were washed with PBS and incubated for two hours with anti-γH2AX (1:300 dilution, Upstate Biotechnology, Lake Placid, NY, USA), followed by incubation with FITC-conjugated anti-mouse IgG (1:100 dilution) for one hour. Slides were immersed in 0.05 mg/ml DAPI for five minutes and then mounted with cover slips using ProLong^® ^Antifade Kit (Molecular Probes Inc., Eugene, OR, USA). Slides were viewed with a Nikon Eclipse E400 fluorescence microscope (Nikon Inc., Tokyo, Japan) and the images were captured by a digital camera and analyzed using SPOT advanced software (Version 4.6, Diagnostic Instrument Inc., Sterling Heights, MI, USA). The number of γH2AX-foci per cell was counted and determined in at least 70 cells for each group [18].

### Single cell gel electrophoresis assay

To compare the degree of DNA fragmentation, mammary tumor cells were pretreated with 40 μM T-oligo or control-oligo for 24 hours, and then subjected to irradiation. Three hours after radiation, cells were trypsinized to single-cell suspension and adjusted to the concentration of 1 × 10^5 ^cells/ml. Cell suspensions (10 μl) and 1% low melting-point (LMP) agarose (100 μl) were gently mixed at 37°C and added onto each CometSlide™ (Trevigen Inc., Gaithersburg, MD, USA). The slides were gelled at 4°C in the dark for 30 minutes, and then immersed in prechilled lysis solution (2.5 M NaCl, 100 mM EDTA, 10 mM Tris) for 60 minutes. Then the slides were immersed in freshly prepared alkaline buffer (300 mM NaOH, 1 mM EDTA, pH > 13.0) for 60 minutes to allow the DNA to unwind prior to electrophoresis at 1 Volt/cm for 30 minutes at 4°C. Air-dried slides were stained for five minutes with 10 μl DAPI (0.05 mg/ml), and then rinsed in cold water and covered with a cover slip. The nuclei were analyzed by use of a fluorescence microscope. Hydroxyl radical-induced DNA damage by H_2_O_2 _was used as a positive control. TriTek CometScore™ (Version 1.5.2.6, Sumerduck, VA, USA) software was used to measure the percentage of DNA in tail. At least 45 cells on each slide were measured.

### β-galactosidase staining

Mammary tumor cells were cultured in either medium alone or medium containing T-oligo or control T-oligo at a concentration of 40 μM for 24 hours, and then irradiated with 3 Gy. Twenty-four hours after radiation, the cells were washed in PBS and fixed with formaldehyde/gluteradlehyde solution (5.6% formaldehyde, 0.4% gluteraldehyde in PBS) for 10 minutes at room temperature and stained with an X-gal mixture (40 mM Citrate/Na_2_HPO_4_, 5 mM K_4_Fe(CN)_6_, 5 mM K_3_Fe(CN)_6, _150 mM NaCl, 2 mM MgCl_2, _1 mg/ml X-Gal) [19] for 24 hours at 37°C. To quantify the β-gal positive cells, three to five high-power fields per mouse were selected and β-gal positive cells were counted as described [19,20] by two investigators.

### TUNEL assay

To determine apoptosis in tumor cells treated with T-oligo and radiation, TUNEL staining was performed using ApopTag Plus peroxidase kit (Chemicon, Temecula, CA, USA) in a method previously described with some modification [21,22]. To quantify the apoptotic cells, three to five high-power fields per mouse were selected and apoptotic cells were counted by two investigators.

### Tumorigenesis of cancer cells treated with T-oligo and radiation

To assess the growth potential of T-oligo and IR-treated tumor cells in mice, mammary tumor cells were supplemented in culture with T-oligo for 24 hours, and then irradiated with 3 Gy. The tumor cells cultured in medium alone or containing control-oligo were used as control groups. After radiation, the cells were washed and prepared for injection. Untreated cells were resuspended in PBS, while the control-oligo and T-oligo-treated cells were resuspended in 1.2 mM control-oligo or T-oligo in PBS. T-oligo or control-oligo-treated and medium only (diluent)-treated tumor cells (1 × 10^6 ^cells in 0.1 ml volume/mouse) were injected respectively in the right or left flanks of syngeneic wild-type mice. Untreated tumor cells with or without IR were also injected as controls. The mice were followed for up to 30 days after the tumor inoculation and tumor growth was measured with calipers every two days. The tumor incidence was also recorded.

### *In vivo *treatment

MMT mice at approximately 73 days of age were injected intraductally in the right chest mammary gland with 50 μL of a T-oligo solution (210 μg = 60 nmol in 50 μL of PBS). The left chest mammary gland in the same mouse was injected with the same dosage of control-oligo. After seven to eight daily injections, the mice were sedated with intraperitoneal administration of ketamine (75 mg/kg) and xylazine (5 mg/kg) and placed in a special Irradiation Pie cage. The mammary glands (chest area) were irradiated with 3 Gy IR with the rest of the body covered by lead foil. The mice were sacrificed 10 days after the radiation. The treated mammary glands were then removed, whole-mounted, formalin-fixed, stained in carmine alum, and photographed with a digital camera. To quantify the tumor burden, digital images of all whole mounted mammary glands were analyzed using SPOT advanced software.

### Statistical analysis

Statistical significance was determined using Student's *t*-tests or *X^2^*-test. One way-ANOVA was used for analysis of data with more than two subgroups.

## Results

### Sensitization of tumor cells to radiation by T-oligo

**Figure 1:**
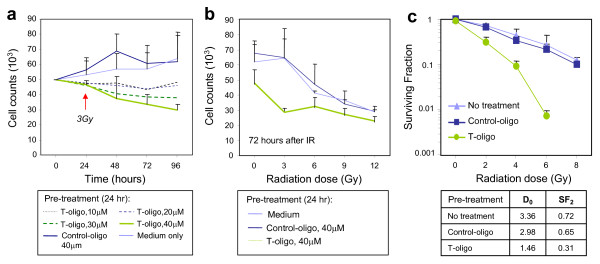
**Inhibition of mammary tumor cell growth by T-oligo and ionizing radiation**. **(a) **Mammary tumor cells isolated from MMT mice were cultured with T-oligo at the concentration of 10, 20, 30 and 40 μM for 24 hours. Tumor cells cultured with control-oligo at the concentration of 40 μM or medium alone were used as control. On Day 2, the cells were exposed to 3 Gy IR. The cells were then collected at indicated time after radiation and counted in triplicates. **(b)** MMT tumor cells were cultured with T-oligo or control-oligo at a concentration of 40 μM for 24 hours and then irradiated with indicated dose of IR or mock irradiated (0 Gy). Seventy-two hours after IR, the cells were counted using a particle counter in triplicates. Statistical significance from three independent experiments was determined by one-way ANOVA. **(c)** Surviving fraction. The tumor cells were cultured in 10 cm tissue culture plates and pretreated with T-oligo or control-oligo at a concentration of 40 μM for 24 hours, and then irradiated with indicated doses. After two-weeks culture, the tumor colonies were fixed and stained with 0.5% crystal violet. The colonies were counted with a cut-off of 50 viable cells. Surviving fractions were calculated by the number of colonies divided as the number of seeded cells × plating efficiency.

To determine if T-oligo can enhance the inhibition of mammary tumor cell growth due to radiation, tumor cells from MMT mice were cultured with T-oligo at concentrations ranging from 0 to 40 μM for 24 hours and then irradiated. Tumor cells cultured in DMEM medium alone or DMEM containing the same concentration of a control oligo were used as controls. The cells were collected and counted at 0, 24, 48, 72 and 96 hours after exposure to 3 Gy IR. T-oligo alone inhibited growth of tumor cells in a dose dependent manner, with the most marked inhibition at 40 μM T-oligo (data not shown). However, inhibition was significantly enhanced after IR and significant inhibitory effects were observed in tumor cells treated with 30 to 40 μM T-oligo and 3 Gy IR (Figure 1a). This group of tumor cells declined in numbers while the control-oligo-treated tumor cells, though exposed to the same dose of 3 Gy, continued to replicate (Figure 1a). These results suggest enhanced inhibition of mammary tumor cells treated with T-oligo and 3 Gy radiation. The inhibition of growth after 40 μM T-oligo and 3 Gy IR was more pronounced than in cells treated with control-oligo or diluent alone (*P *= 0.019) and 6, 9 or 12 Gy (Figure 1b).

The clonogenic cell survival assay is the gold standard to measure the radiosensitivity of cells. To determine if T-oligo can sensitize mammary tumor cells to radiation, tumor cells pretreated for 24 hours with T-oligo, control-oligo or medium alone were irradiated with indicated doses of radiation and then the surviving fraction of cells (those capable of colony formation) was determined. As shown in Figure 1c, the survival curve of tumor cells treated with T-oligo and IR shifted to the left considerably compared with those treated with control-oligo or medium and 3 Gy, suggesting significant radiosensitization of tumor cells by T-oligo (Figure 1c). The mean lethal dose (D_0_) for tumor cells treated with radiation plus T-oligo, control-oligo or medium alone was 1.46, 2.98 and 3.36 Gy, respectively (Figure 1c). The survival fraction at 2 Gy (SF_2 _= 0.31) for tumor cells treated with T-oligo and radiation are comparable to those of cell lines considered to be radiosensitive [23]. Thus pretreatment with T-oligo increases the sensitivity of tumor cells to radiation and the combined treatment with T-oligo and radiation can lead to cell growth arrest and/or death as demonstrated by the clonogenic assay.

### Mechanism of T-oligo-induced hypersensitivity to radiation

**Figure 2:**
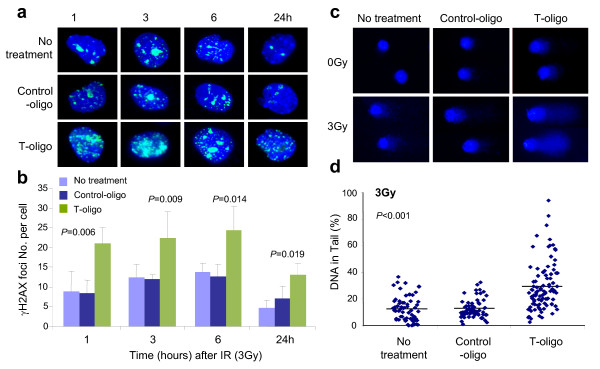
**Assessment of DNA damage responses and DNA repair**. **(a, b) **Immunofluorescence staining for γH2AX. Tumor cells isolated from MMT mice were cultured with T-oligo or control-oligo at a concentration of 40 μM for 24 hours in chamber slides. After 3 Gy IR, cells were stained with FITC-anti-γH2AX mAb at indicated time. The number of γH2AX foci per cell was counted and calculated from at least 70 cells in each group after subtraction of γH2AX foci from the un-irradiated tumor cells and presented as means ± SD from repeated experiments. Statistical significance was determined by one-way ANOVA. **(c, d) **Comet assay. Tumor cells treated with T-oligo or control-oligo (40 μM) or medium with or without 3 Gy IR 24 hours later were assessed for DNA repair at three hours after IR using Trevigen's CometAssay^® ^kit. Comet images were selected randomly from the microscope field of view using a defined sequence of searching to ensure the same image was only scored once. Statistical significance was determined by one-way ANOVA.

To investigate potential mechanisms behind radiosensitization of tumor cells by T-oligo, we next examined the levels of nuclear-foci containing phosphorylated H2AX (H2AX), a modification that occurs at sites of DNA breaks [24]. Figure 2a shows representative immunofluorescent images of γH2AX foci in treated mammary tumor cells. Increased numbers of γH2AX foci per cell were observed in tumor cells treated with T-oligo and 3 Gy IR at every time point after radiation compared with those in tumor cells treated with control-oligo or medium alone and 3 Gy (Figure 2a, b). The difference in γH2AX-focus number between tumor cells treated with T-oligo and IR and control groups at 1, 3, 6 and 24 hours is statistically significant (Figure 2b). These results suggest that T-oligo enhances radiation-induced DNA damage signal and/or delays DNA repair, although T-oligo alone is known to transiently induce γH2AX foci at telomeres in the apparent absence of double strand DNA breaks or other damage [25]. We next compared DNA fragmentation in tumor cells treated with T-oligo and 3Gy IR using the comet assay [26]. Very modest DNA fragmentation indicated by percentage of DNA in tails, was observed in tumor cells treated with T-oligo or 3 Gy IR alone, while cells treated with control-oligo or medium alone showed no fragmentation (Figure 2c, d). However, a significantly greater amount of fragmented DNA was detected three hours after 3 Gy IR treatment in T-oligo-pretreated cells (Figure 2c, d). Thus, combined treatment with T-oligo and radiation results in enhanced response to DNA damage and/or impaired DNA repair, leading to growth arrest and cell death.

### Increased induction of senescence and apoptosis in tumor cells pretreated with T-oligo

**Figure 3:**
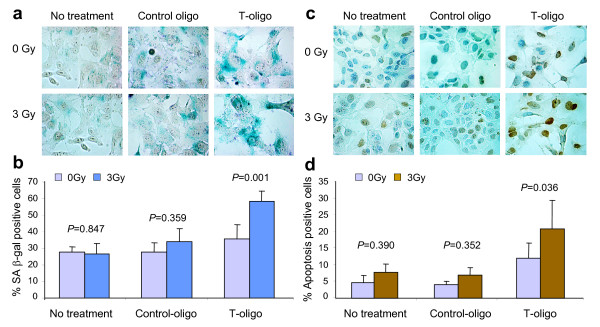
**Increased senescence and apoptosis in tumor cells treated by T-oligo and IR**. Mammary tumor cells isolated from MMT mice were treated with 40 μM T-oligo, Control-oligo or medium (diluent) alone for 24 hours and then irradiated. Cells cultured in medium alone were used as control. The tumor cells were collected 24 hours after 3 Gy IR and assayed for senescence and apoptosis. **(a, b) **β-gal staining. (a) The cells stained blue are β-gal positive cells (magnification 60×). (b) Percentage of β-gal positive cells in T-oligo or control-oligo-treated dishes with or without IR is presented in a bar graph. **(c, d) **TUNEL staining. (c) The cells stained in brown color are apoptotic cells (magnification 60×). (d) Percentage of apoptotic positive cells in T-oligo or control-oligo-treated tumor cells with or without IR is presented in a bar graph. Statistical significance from repeated experiments was determined by *X^2^*-test.

As the above studies show that cells pretreated with T-oligo are more sensitive to IR, we next determined whether the treated cells undergo senescence or apoptosis. Tumor cells isolated from MMT mice were pretreated with T-oligo or control-oligo followed by radiation and then examined 24 hours later for the induction of senescence or apoptosis using senescence-associated β-galactosidase (S.A. β-gal) and TUNEL staining, respectively. Increased numbers of large (flattened) cells positive for S.A. β-gal, two markers of senescent cells, were observed after T-oligo treatment compared with control-oligo treatment or medium alone. However, β-gal-positive cells increased significantly in tumor cells treated with T-oligo and 3 Gy compared with control-oligo or no treatment and 3 Gy (58.3 ± 6.3% vs 34.2 ± 7.5% or 26.1 ± 6.2%, Figure 3a, b). A more profound effect of combined T-oligo and IR was detected using the TUNEL assay. The apoptotic rate increased significantly to 20.8 ± 8.5% (*P *= 0.036), two to four times the control rates, in tumor cells treated with T-oligo and 3 Gy (Figure 3c, d). These results indicate that senescence and apoptosis may be important pathways to inhibit proliferation of murine mammary tumor cells treated with T-oligo and IR. Both responses may also contribute to the observed decrease in clonogenic ability. Given that rates of senescence and apoptosis have been previously observed to increase steadily in T-oligo-treated malignant cells over two to four days, depending on cell type [5-8], these determinations made only 24 hours after irradiation, preceded by an overnight T-oligo incubation, may underestimate the eventual impact of the treatment.

### Decreased tumorigenesis in mammary tumor cells treated with T-oligo and irradiation

**Figure 4:**
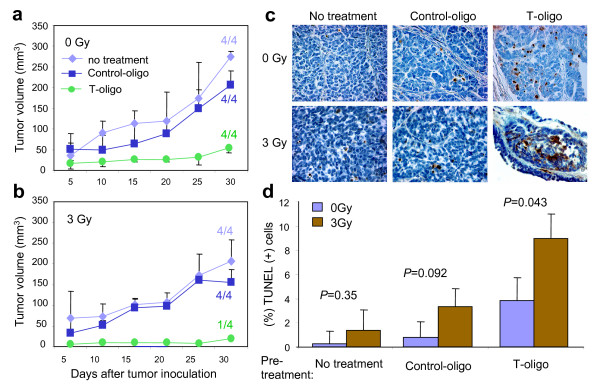
**Loss of tumor forming ability in mammary tumor cells treated with T-oligo and radiation**. **(a, b) **Mammary tumor cells were cultured in medium containing 40 μM T-oligo, control-oligo, or no additive. After 24 hours of culture, the tumor cells from each group were collected, counted, placed in the 50 ml polypropylene tube on ice and then irradiated with 0 or 3 Gy, respectively. Then 1 × 10^6 ^tumor cells from each group with or without irradiation were injected subcutaneously into right or left flanks of syngeneic wild-type mice (*n *= 4/group). Mice (*n *= 4/group) injected with tumor cells cultured with medium alone and/or that received radiation alone were used as controls. Tumor size was measured with calipers every other day for 30 days and tumor volume was calculated using the formula: tumor volume = (longer diameter × shorter diameter^2^)/2. The average tumor volume was calculated and presented at indicated days after tumor inoculation. The tumor incidence was indicated as the numbers of tumor-bearing mice/total numbers of mice in each group in the graph. Statistical significance was determined by one-way ANOVA. **(c) **Sections of tumors from mice inoculated with tumor cells treated *in vitro *with T-oligo or control-oligo followed by 0 or 3 Gy radiation were stained for apoptosis via TUNEL assay and then photographed at 40× magnification. **(d) **The cells stained brown are apoptotic. The apoptotic cells were counted in three to five randomly selected fields at 40× magnification. The percentage of apoptotic cells among all tumor cells is graphed and statistical significance was determined by *X^2^*-test.

To determine whether tumor cells can still form tumors *in vivo *after treatment with T-oligo and radiation, mammary tumor cells were preincubated with T-oligo or control-oligo for 24 hours followed by exposure to 0 Gy (mock) or 3 Gy. The other control groups were tumor cells supplemented with medium (diluent) alone or exposed to 3 Gy of radiation alone. The tumor cells (1 × 10^6^) were then injected subcutaneously into the flanks of syngeneic wild-type mice. As shown in Figure 4a, all mice inoculated with tumor cells pretreated with T-oligo alone developed tumors, but tumor size was reduced when compared with untreated and control-oligo-treated tumor cells on Day 30 (Figure 4a, *P *= 0.02). However, the tumor forming ability of mammary tumor cells treated with combined T-oligo and 3 Gy irradiation was almost eradicated. Only one out of four mice developed a tumor and this small tumor did not appear until after Day 25 (Figure 4b). The difference in volume for tumor arising from cell populations treated with T-oligo vs control-oligo or medium alone, followed by 3 Gy, was highly statistically significant (*P *= 0.001, Figure 4b). Next, to determine if T-oligo and radiation induce apoptosis in tumor cells injected into mice, sections of tumors from all groups were stained for apoptosis using the TUNEL assay. In tumors arising from cells pretreated once with T-oligo alone we observed far more apoptosis even after 30 days compared with control-oligo or medium alone (Figure 4c). Few or no TUNEL-positive cells were observed in tumors arising from control-oligo or medium treated cells, and 3 Gy irradiation did not increase the apoptosis to a statistically significant degree (Figure 4c, d). In tumors arising from cells treated with combined T-oligo and radiation, the numbers of apoptotic cells increased significantly (*P *= 0.043) even after 30 days. At this time, an approximately three- to six-fold increase in apoptosis was observed in tumor cells treated with T-oligo vs control-oligo or medium alone prior to subcutaneous injection (Figure 4d). The one small tumor found in one mouse inoculated with tumor cells treated with T-oligo followed by 3 Gy IR contained many apoptotic cells (Figure 4c, bottom right panel). These experiments provide further evidence that pretreatment with T-oligo can enhance the apoptotic killing of tumor cells by radiation, even by radiation doses that alone have only a modest effect.

### Effect of combined T-oligo and radiotherapy on spontaneous mammary carcinomas *in vivo*

**Figure 5:**
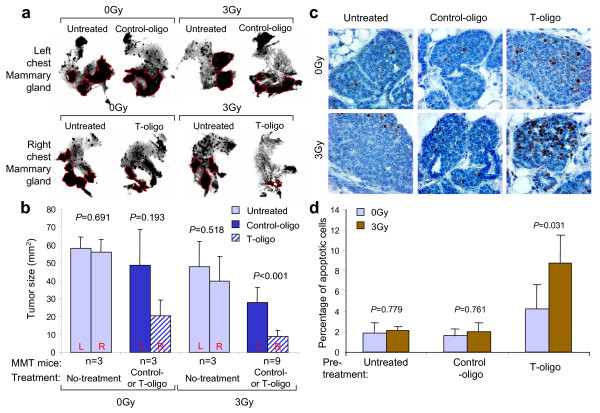
**Effect of T-oligo and radiation therapy on spontaneous mammary carcinomas *in vivo***. **(a) **Photos of whole-mounted mammary glands. Panels show representative mammary gland/tumor harvested from mice injected with control-oligo (left side) and T-oligo (right side), respectively. After seven to eight injections, the mammary glands (chest area) of oligo-treated mice (*n *= 9) and untreated mice (*n *= 3) were irradiated with 3 Gy after being anesthetized with intraperitoneal administration of ketamine (75 mg/kg) and xylazine (5 mg/kg). The remaining oligo-treated and untreated mice (*n *= 3) that received no IR were used as control. The mice were sacrificed on Day 10 after irradiation and mammary glands were harvested, processed for whole mount and photographed. The dark nodules circled by red line are the mammary tumors. **(b) **Comparison of tumor size from the treated and control mice. The whole mount was digitized and the tumors were traced and measured using SPOT advanced software. Statistical analysis was performed using *t*-test. Untreated right (R) and left (L) chest mammary glands in mice receiving either 0 Gy or 3 Gy, were harvested and tumor size is presented separately to show the minimal and insignificant site variation. **(c) **TUNEL staining. The mammary tissues in panel A were fixed, sectioned and stained for apoptosis (60×). **(d) **The apoptotic (brown) cells were counted by two investigators and the percentage of apoptotic cells among each group (*n *= 3/group) was calculated and presented in bar graph. Statistical significance was determined by *X^2^*-test.

When evaluating the efficacy of a therapy for breast cancer, it is desirable to use a tumor model that resembles breast cancer in humans as closely as possible. We [16,27] and others [15,28] have demonstrated that PyMT-induced mammary tumors share many features in common with human breast cancer. Therefore, MMT mice were used to evaluate the combined effect of T-oligo and radiotherapy in the *in vivo *setting. Our previous studies showed that mammary tumors in MMT mice at Days 70 to 80 are in the early invasive stage [16]. MMT mice (*n *= 9) aged 70 to 72 days received intraductal injections of T-oligo at a dose of 210 μg in 50 μL PBS every other day in a right chest mammary gland. A left chest mammary gland was injected with the same dose of control-oligo as a same animal control. After seven to eight injections, the mice were irradiated with a single dose of 3 Gy focused on the chest area. The control groups consisted of three littermates each that received no treatment, treated with T-oligo and control-oligo without radiation or treated with 3 Gy IR alone. Ten days after irradiation, the mice were sacrificed and the treated and control mammary glands harvested, processed for whole mount, and digitized. The tumor in the digital image was traced and analyzed by SPOT advanced software. As shown in Figure 5a, b, mammary tumors in the mice without treatment or treated with 3 Gy alone reached the size of 58 ± 6.11 mm^2 ^and 48 ± 14.18 mm^2^, respectively, statistically comparable. Treatment with T-oligo or control-oligo without irradiation resulted in tumor sizes of 20.3 ± 8.96 mm^2 ^and 48.6 ± 30.1 mm^2^, respectively. However, the average tumor size in the right mammary gland treated with T-oligo and 3Gy IR was reduced to 8.67 ± 3.61 mm^2^, compared with the average tumor size of 27.67 ± 8.69 mm^2 ^in the left mammary gland treated with control-oligo and 3 Gy IR. This reduction in tumor size was highly statistically significant (*P *< 0.001). The reduction in tumor size is more than the combined reduction by the treatment of T-oligo or radiation alone when compared with those in the no treatment group. Consistent with the observed reduction in tumor size, there was a striking increase in TUNEL positive cells 10 days post-irradiation in T-oligo treated tumors (Figure 5c, d). Taken together, these results indicate that an additive or synergistic effect of T-oligo and radiation therapy can be achieved in a murine model that is closely related to breast cancer in humans.

## Discussion

Ionizing radiation induces both single- and double-strand DNA breaks (DSB) in cells that then trigger DNA damage responses characterized by the recruitment of DNA-repair proteins to γH2AX foci at sites of DNA damage and the activation of checkpoint proteins that arrest cell cycle progression [29]. Cell cycle arrest is a protective cellular response understood to block cell cycle progression and to permit DNA damage repair [29]. An increase in DNA damage, reduced ability to repair DNA damage, and/or prolonged checkpoint activation can cause apoptosis [30] or cause cells to undergo permanent cell cycle arrest (senescence) [31]. We show here that pretreatment with T-oligo sensitizes mammary tumor cells to radiation, promoting growth inhibition and death of tumor cells *in vitro *and in an *in vivo *mouse model.

The mechanism by which T-oligo sensitize tumor cells remains to be fully elucidated. Although T-oligos do not act as telomerase inhibitors [12] or cause digestion of the 3' telomere overhang [5,25,32], T-oligos have been shown to rapidly concentrate in nuclei when added to cultured cells and the subsequent responses require WRN [25], the protein mutated in the progeroid cancer-prone Werner syndrome. T-oligo/WRN interaction results in formation of DNA γH2AX damage-like foci at the telomeres [25] with activation of ATM [8,32] and its many downstream effector proteins, leading to apoptosis and senescence [5,7,8,32]. In the present study, pretreatment with T-oligo enhances the formation of γH2AX foci (Figure 2) that customarily form at sites of DNA damage but after T-oligo treatment form at telomeres in the absence of detectable DNA damage [25]. Activating the DNA damage response pathways by T-oligo treatment, as demonstrated to occur over several days in multiple cell types including breast carcinoma cells [5-7,9], could render tumor cells more apt to undergo apoptosis or senescence when exposed to IR. Alternatively, tumor cell inactivation could be due to the impairment of DNA repair by the pretreatment with T-oligo as demonstrated by slower decay of γH2AX foci and increased fragmentation of DNA in the comet assay (Figure 2). Increased radiosensitivity has been found in cells from patients with DDR or DNA-repair disorders such as Ataxia Telangiectasia (defect in ATM), Nijmegen Breakage Syndrome (defect in NBS1), Fanconi anemia, defective Artemis, DNA ligase I and DNA ligase IV [33]. We favor a model of radiosensitization by T-oligos that encompasses the known and hypothesized effects of both IR and T-oligos: After 24 hours pretreatment, T-oligo-treated cells have entered an S-phase arrest [5-8,11] mediated by p95/Nbs1 [10], presumptively due to G-quadruplex formation between single stranded telomeric DNA and the G-rich T-oligos [12] with consequent stalling of replication forks [34]. Without further intervention, malignant cells then begin to undergo apoptosis [5-8,11] or to enter senescence [8,35] or both, as in the case of breast carcinoma cells in this study and a previous one [8], presumably in response to the collapse of their stalled replication forks. If such cells are then irradiated, the introduction of even modest numbers of DSBs and other DNA damage greatly enhances the replication stress and the processes of apoptosis and senescence.

Regardless of its mechanism of action, T-oligo pretreatment increased tumor cell sensitivity to radiation as demonstrated by the clonogenic assay (Figure 1). The present data suggest that combining T-oligos with low dose IR may permit safer and more effective radiotherapy of breast cancer and potentially other malignancies. T-oligo adjuvant therapy would thus be very beneficial to patients otherwise at risk of short-term and long-term adverse effects of IR, including radiation dermatitis, fibrosis, compromised wound healing, and secondary malignancies [36]. T-oligos when applied alone are without detectable adverse effects on normal tissues after either local or systemic administration in multiple mouse models [6,8,9,11,12] including the MMT mice. In accordance with this, we did not observe adverse effects in mice exposed to this agent and 3Gy IR including lethargy, anorexia, inactivity, ruffled fur coat or diarrhea.

The murine mammary tumor induced by PyMT shares many features with poor-prognosis human breast cancer such as a high frequency of distant metastases, persistent expression of biomarkers, ErbB2/Neu and cyclin D1, and loss of estrogen and progesterone receptor expression [28]. In addition, the tumors develop in multiple stages amid a competent immune system, a trait also shared by human breast cancer [22]. These advantages would appear to outweigh the greater individual variation in mammary tumor development in MMT mice versus mice bearing xenografts of established breast cancer cell lines. MMT mice thus provide a reliable model for the study of tumorigenesis in breast cancer as well as a useful tool for the evaluation of treatment modalities.

## Conclusions

In this study, we demonstrated that pretreatment with T-oligo sensitizes mammary tumor cells to radiation *in vitro *and *in vivo *tumor models. The inhibition of tumor cells by pretreatment with T-oligo was associated with increased induction of senescence and apoptosis of irradiated tumor cells and reduced clonogenesis, presumably due to the observed increased formation and/or delayed resolution of DNA damage response foci. Further studies of combined T-oligo/IR therapy are warranted.

## Abbreviations

β-gal: β-galactosidase; γH2AX: phosphorylated histone H2AX; DDR: DNA damage response; DSB: double-strand DNA breaks; IR: Ionizing radiation; PyMT: polyomavirus middle T oncogene; Tg: transgenic; T-oligos: oligonucleotides homologous to the telomere G-rich sequence TTAGGG; TUNEL assay: terminal deoxynucleotidyl transferase dUTP Nick End Labeling assay.

## Competing interests

Two co-authors, Mark Eller and Barbara Gilchrest, have equity in the for-profit start-up company SemaCo and a patent related to the content of the manuscript. Barbara Gilchrest is Semaco's Chief Scientific Officer.

## Authors' contributions

DW participated in the design of the study, carried out clonogenic cell survival and comet assays, immunofluorescence staining, T-oligo treatment and radiation experiments, and performed the statistical analysis. MCC participated in the design of the study, carried out cell counting, β-gal and TUNEL staining, T-oligo treatment and radiation experiments. BS participated in the design of the study and helped draft the manuscript. BDP, MSE and BAG provided expert views in the radiation experiment and helped revise the manuscript. SKC participated in the drafting of the manuscript. JG conceived of the study, designed, coordinated and participated in the experiments, and drafted the manuscript. All authors read and approved the manuscript.
